# Special Issue “Diagnosis and Treatment of Invasive Pulmonary Fungal Infections”

**DOI:** 10.3390/jof9070744

**Published:** 2023-07-13

**Authors:** Joseph P. Lynch, Dimitrios P. Kontoyiannis

**Affiliations:** 1 Division of Pulmonary, Critical Care, Interventional Pulmonology, Sleep, and Clinical Immunology, David Geffen School of Medicine at UCLA, Los Angeles, CA 90095, USA; 2 Department of Infectious Diseases, Infection Control and Employee Health, The University of Texas MD Anderson Cancer Center, Houston, TX 77030, USA

The Guest Editors Dr. Joseph P. Lynch, III and Dr. Dimitrios P. Kontoyiannis developed this issue of *Journal of Fungi*, focusing on invasive pulmonary fungal infections, to provide a comprehensive, clear, and cogent perspective of these pathogens. World-recognized experts were recruited to write chapters on specific fungi/molds, featuring in-depth discussion of the risk factors, prevalence, clinical features, keys to diagnosis, and therapy. The first three chapters on histoplasmosis, blastomycosis, and coccidiomycosis primarily encompass community-acquired fungi in both immunocompetent and immunocompromised hosts, whereas chapters 4 through 9 discuss fungi/molds that primarily affect immunocompromised individuals. Chapter 10 provides an in-depth review of systemic therapeutic options for invasive pulmonary infections, while chapter 11 focuses on inhaled antifungals for prophylaxis and treatment of fungal pneumonias. We trust that this issue provides superb coverage of relevant issues in the diagnosis and treatment of fungal/mold infections. Below are brief summaries of the chapters in this issue.

## 1. Pulmonary Histoplasmosis: A Clinical Update by Nicolas Barros, Joseph L. Wheat, and Chadi Hage [1]

In the first chapter, Dr. Barros et al. discuss histoplasmosis, a fungal disease due to *Histoplasma capsulatum* that is endemic in certain areas in the USA (i.e., within Mississippi and Ohio River valleys) and some parts of Canada, as well as Southeast Asia, and countries in southern Africa. In recent decades, histoplasmosis outside of the historical areas likely reflects the effects of international travel, increased use of immunosuppressive therapy, and climate change. The lung is the usual site of entry, and the most common site of clinical involvement. The disease’s clinical features are varied, and include acute or chronic pneumonia, chronic pulmonary cavities or nodules, mediastinal lymph node or vascular involvement, fibrosing mediastinitis, and extrapulmonary dissemination. The vast majority of infected immunocompetent hosts are asymptomatic or have mild symptoms that resolve spontaneously. These cases can be monitored without treatment. However, chronic pulmonary disease or extrapulmonary spread requires treatment. Dissemination is ten times more common in immunocompromised hosts, e.g, HIV infection, hematological malignancy, solid organ transplantation, and immunosuppressive therapy (particularly anti-TNFα agents). The authors provide practical and in-depth recommendations for therapy depending on the severity and sites of disease. Moderate-to-severe acute pulmonary histoplasmosis should be treated with lipid AmB for 1-2 weeks, followed by itraconazole (ITRA) for a total of at least 12 weeks. All immunocompromised patients with histoplasmosis should be treated, usually with a short course of lipid AmB followed by ITRA for at least 12 months, alongside those with negative or low histoplasma antigen. 

## 2. Blastomycosis by Kathleen A. Linder, Carol A. Kauffman and Marisa H. Miceli [2]

This chapter represents an outstanding review of blastomycosis, a fungal disease endemic in certain regions in the USA and Canada, primarily around water sources (e.g., the Great Lakes, St. Lawrence Seaway) and in several central and southeastern states in the USA. The vast majority of cases in the genus *Blastomyces* are due to the species *B. dermatitidis.* The lung is the usual site of entry, and the most common site of clinical involvement. Although many infections are asymptomatic, pneumonia and (rarely) acute respiratory distress syndrome (ARDS) can occur. Extrapulmonary dissemination occurs in 20-50% of cases, most commonly in skin and soft tissues, followed by osteoarticular and genitourinary systems. The authors provide a detailed and practical approach to therapy depending upon site, clinical features and response, and the toxicity of therapy. All symptomatic patients should be treated, as untreated cases are at increased risk for progression or recurrence. Mild-to-moderate pulmonary blastomycosis without CNS involvement can be treated with an oral azole (i.e., itraconazole) for 3 to 6 months depending upon clinical and radiological response. Extrapulmonary infections require 6 to 12 months of therapy, and osteoarticular infections a minimum of 12 months. CNS blastomycosis warrants treatment with a lipid formulation of AmB daily for 4 to 6 weeks followed by an oral azole (preferentially voriconazole) for at least one year. 

## 3. Diagnosis and Treatment of Pulmonary Coccidiomycosis and Paracoccidiomycosis by Paula Massaroni Peçanha-Pietrobom, Andrés Tirado-Sánchez, Sarah Santos Gonçalves et al. [3]

This chapter represents an excellent review of coccidioidomycosis (cocci) and paracoccidioidomycosis (PCM), invasive fungal diseases that are endemic in the Americas but quite rare elsewhere in the world. In the Americas, these pathogens are due to either *Coccidioidococcus neoformans* or *C. gattii* complexes. Pulmonary signs and symptoms dominate for these pathogens, but extrapulmonary spread to the skin, viscera, or CNS may occur in patients with depressed T cell immunity. In the Americas, most cases are acquired in dry deserts or near desert regions. The authors discuss this pathogen’s histopathological features (typically spherular with endospores) in tissue or bronchoalveolar lavage fluid, as well as serologies for antibodies, antigen or PCR. The disease is usually self-limited, so treatment is often not necessary in normal hosts with mild disease. Severe pulmonary and extrapulmonary involvement may occur in immunosuppressed individuals. Triazoles (fluconazole or itraconazole) are the standard therapeutic route, but amphotericin B is used in cases that are refractory to triazoles, or in critically ill patients. 

PCM is endemic in Latin America, particularly in Argentina, Brazil, Columbia, and Venezuela. *Paracoccidioides brasiliensis* is the predominant species. PCM is rare outside endemic areas, but cases may occur in individuals who have traveled to Latin America. Interestingly, a chronic form of PCM with pulmonary fibrosis may occur several decades after infection. *Paracoccidiodes* is susceptible to virtually all antifungal agents, as well as trimethoprim–sulfamethoxazole. The agent of choice for mild-to-moderate disease is itraconazole, used for 9 to 12 months. For severe cases, initial treatment with amphotericin B (preferably liposomal) for 2 to 4 weeks is recommended, followed by an azole for 9 to 12 months.

## 4. Invasive Pulmonary Aspergillosis by Marie-Pierre Ledoux and Raoul Herbrech [4]

This chapter presents an outstanding review of molds within the genus *Aspergillus*, which includes multiple species, the most relevant of which are *A. fumigatis* (in Europe the USA) and *A. flavus* (in some Asian countries and elsewhere). *Aspergillus* spores are ubiquitous, found in indoor and outdoor air, soil, decomposing plants, flowers, household dust, and building materials. Invasive aspergillosis (IA) only affects immunosuppressed individuals. The most common risk factors include hematological malignancy, organ transplants, and treatment with high-dose corticosteroids or immunosuppressive agents. The lungs are the primary site of involvement, but extrapulmonary spread or dissemination can occur in severely immunocompromised individuals. Chest radiographs or CT scans may show multiple or single nodules, ground glass opacities, or focal consolidation. *Aspergillus* may also cause allergic bronchopulmonary aspergillosis (ABPA), a noninvasive allergic infection, or fungus balls in the lungs (mycetomas). Galactomanan (GM) is a polysaccharide component of *Aspergillus* cell walls, and detection of GM in serum, bronchoalveolar lavage fluid, or cerebral spinal fluid is more sensitive than in cultures. Although amphotericin-B (AmB) deoxycholate was the treatment of choice for many years, voriconazole or isavuconazole are less toxic and just as (if not more) effective, and are considered first line agents. For individuals intolerant of azoles, liposomal AmB can be substituted. Because IA is associated with high mortality and recurrent rates, treatment should be continued for a minimum of 6 to 12 weeks, and longer for individuals showing less than a complete response.

## 5. Pulmonary Cryptococcosis by Annaleise R Howard-Jones, Rebecca Sparks, David Pham et al. [5]

Cryptococcosis is an invasive mycosis caused by yeasts principally of the *Cryptococcus neoformans* or *Cryptococcus gattii* species complexes. Cryptococcosis occurs more commonly in immunosuppressed hosts. *Cryptococcus* is usually acquired via inhalation into the respiratory tract of desiccated yeast in vegetation and soils, or via exposure to avian guano. Pulmonary involvement is the most common manifestation, but the involvement of the CNS (meningitis, encephalitis) may be devastating. *Cryptococcus neoformans* is the predominant species worldwide, but *C. gattii* typically occurs in regions such as New Guinea, Australia, and parts of the USA and Canada. The authors discuss distinctions in clinical features between *C. gattii and C. neoformans*, the risk factors for acquisition, and diagnosis via serological, histological, and culture techniques. Therapy of cryptococcosus involves an induction phase with amphotericin B and 5-flucytosine for 2 to 4 weeks, followed by consolidation and maintenance phases with oral fluconazole for at least a year. Adjunctive surgical debridement may be required for cases with extensive disease or large focal masses. Mortality may exceed 50% in immunocompromised hosts or cases with dissemination or meningoencephalitis.

## 6. What Is New in Pulmonary Mucormycosis? by François Danion, Anne Coste, Coralie Le Hyaric et al. [6]

Mucormycosis (MCR) is a rare but life-threatening infection due to molds of the order *Mucorales*. The rhino-orbito-cerebral (ROC) region is the most common site, followed by the pulmonary soft tissues and skin. Major risk factors include hematological malignancies, organ transplantation, severe neutropenia, immunosuppression, corticosteroids, and poorly controlled diabetes mellitus (DM). Hematological malignancy is the most common risk factor associated with pulmonary MCM, whereas diabetes mellitus (DM) is most often associated with ROC. The incidence of MCR has increased over the past three decades, but is still 10 to 50 times less common than invasive candidiasis or aspergillosis. Its incidence is 70 times more common in India than in the rest of the world, likely reflecting a high frequency of DM, malnutrition, and colonization of soil. Additionally, a dramatic increase in MCR was observed in India, beginning in 2021, in patients infected with COVID-19. The diagnosis of MCR is difficult, as cultures are (+) in only 15 to 25% of cases. Molecular methods and/or histology are important to substantiate the diagnosis. Liposomal amphotericin B is the first-line therapy, but surgical resection or debridement may be critical as an adjunctive therapy. After stable or partial response, step-down treatment includes oral isavuconazole or posaconazole until complete response. Correction of neutropenia (or minimization of immunosuppressive therapy) is critical to improve outcomes. Mortality associated with MCR is high (30-90%) depending upon the involved site and presence or absence of dissemination. 

## 7. Non-Aspergillus Hyaline Molds (*Scedosporium*, *Fusarium*, *Scropulariopsis*, *Penicillium*) by Samantha E. Jacobs and Thomas J. Walsh [7]

In this chapter, the authors astutely discuss sinopulmonary infections due to non-*Aspergillus* hyaline molds (e.g., *Fusarium*, *Scedosporium*, *Lomentospora prolificans*, *Paecilomycetes*, *Trichoderma*, and others). Hyaline molds have nonpigmented septate hyphae with acute angle branching. These infections occur primarily in severely immunocompromised individuals (e.g., prolonged neutropenia, organ transplant recipients, cystic fibrosis, chronic immunodeficiency syndrome), and rarely in healthy individuals, following trauma, burns, near drowning, or iatrogenic exposure. Use of antifungal prophylaxis may select for these highly resistant fungi. The authors comprehensively discuss the risk factors associated with hyaline mold infections, their salient clinical features, and therapeutic approaches. Optimal medical therapy requires the determination of susceptibility to antimicrobial agents(s), as many species are resistant to amphotericin B and/or azoles. In addition to treatment with antifungal agents, surgery or debridement may have important adjuvant roles. Reversal of immunosuppression or neutropenia and restoration of immunity are critical to a favorable outcome.

## 8. *Pneumocystis jirovecii* by Anna Apostolopoulou and Jay A. Fishman [8]

*Pneumocystis jirovecii* (formerly termed *Pneumocystis carinii*) is an important cause of pneumonia in severely immunized hosts (e.g., HIV infection, organ transplantation, hematological malignancy, high-dose corticosteroids or immunosuppressive therapy). The authors provide an outstanding history of the recognition of this pathogen, initially among undernourished children in the 1940s and 1950s, followed by cases in individuals with hematological malignancy and in organ transplant recipients (especially allogeneic hematopoietic stem cell recipients) in the 1970s, and HIV-infected patients in the 1980s. The authors discuss the pathogenesis of *Pneumocystis* infections in humans and animal models, and emphasize the key role of impaired immunity (due to high-dose corticosteroids, immunosuppressive agents, CD4 lymphocyte depletion, rituximab, alemtuzumab, etc.) in facilitating infection. Trimethoprim/sulfamethoxazole has proved to be a highly efficacious form of therapy, and its use as prophylaxis in high-risk patients has dramatically reduced the incidence of serious infections due to this pathogen.

## 9. Invasive Fungal Infections Associated with COVID-19 by Kyaw M. Hlaing, Lea M. Monday, Marcio Nucci et al. [9]

Severe COVID-19 with lung injury has been associated with a markedly increased incidence of opportunistic fungal infections [e.g., invasive pulmonary aspergillosis (IPA), candidiasis, mucormycosis (MCR), and cryptococcosis), particularly in patients admitted to the ICU and requiring endotracheal intubation (20-35% incidence). The use of immunomodulators (e.g., baricitinib and oxcilizumab) and immunosuppression agents (e.g., dexamethasone) for cytokine storm syndrome in severe COVID-induced lung disease alters cytokine and chemokine release, and predisposes individuals to invasive fungal infections (especially IPA). Voriconazole (VORI) has excellent activity against *aspergillus* for COVID-19-associated pulmonary aspergillosis (CAPA); however, multiple drug interactions as well as mild interaction with some COVID-19 treatments (i.e., remdesivir) may be problematic. Isavuconazole has fewer drug interactions and more favorable pharmacokinetics. When triazoles (voriconazole or isavuconazole) cannot be used for the treatment of CAPA, liposomal amphotericin B is an option. Early in 2020, several cases of candidemia were reported globally in patients with COVID-19 pulmonary infections; many had received tocilizumab. Most patients were on mechanical ventilation, vasopressors, antibiotics, corticosteroids (CS), parenteral nutrition, and had central venous catheters. From 2020, COVD-associated mucormycosis (CAM) has been described in India, the USA, and Europe. In India, most cases of CAM have had rhino-orbital (RO) involvement, whereas elsewhere, pulmonary involvement has proved to be the most common. CAM has been associated with uncontrolled diabetes mellitus, the use of CS, and severe illness.

## 10. Systemic Antifungal Therapy for Invasive Pulmonary Infections by Ronen Ben-Ami [10]

This fantastic paper reviews the three available classes of anti-fungal agents (polyenes, azoles, echocandins) in a comprehensive and highly practical way. It discusses the mechanisms of action of the various agents, their susceptibility, efficacy, evolution of resistance, and adverse effects, in comparison to other agents. Extensive references enable the reader to easily identify the strengths and weaknesses of specific agents, and the most appropriate agents to use for specific fungi. 

## 11. The Role of Inhalational Antifungals for Prophylaxis and Treatment of Fungal Pneumonia by Nancy N. Vuong, Danielle Hammond, Dimitrios P. Kontoyiannis [11]

This chapter details the role of inhaled antifungal agents in the prophylaxis or adjunctive treatment of patients at high risk of invasive fungal or mold infections (i.e., prolonged neutropenia, hematological malignancies, organ transplant recipients (especially lung), and recent infections with COVID-19), which is controversial. Liposomal amphotericin B 12.5 mg inhalation once weekly may be an option, but data are limited, and no consensus exists regarding the role of inhaled antifungal agents. Data regarding other agents (e.g., azoles) are scant. The authors performed a thorough review of the literature and emphasize the need for prospective, randomized trials to address this issue.
